# Cascading Effects of Overhunting on the Functional Tree Composition of Amazonian Forests

**DOI:** 10.1002/ece3.72657

**Published:** 2025-12-17

**Authors:** Andressa Bárbara Scabin, Iago Ferreiro‐Arias, Flávia Regina Capellotto Costa, Ana Benítez‐López, Cintia Gomes de Freitas, Carlos A. Peres

**Affiliations:** ^1^ Pós‐Graduação em Ecologia Universidade Federal do Rio Grande do Norte—Natal Natal Brazil; ^2^ Instituto Juruá Manaus Amazonas Brazil; ^3^ Department of Conservation Biology and Global Change Estación Biológica de Doñana (EBD‐CSIC) Sevilla Spain; ^4^ Department of Biogeography and Global Change Museo Nacional de Ciencias Naturales (MNCN‐CSIC) Madrid Spain; ^5^ Coordenação de Biodiversidade Instituto Nacional de Pesquisas da Amazônia Manaus Amazonas Brazil; ^6^ Laboratório de Ecologia Vegetal, Instituto de Biologia Universidade Federal do Rio de Janeiro Rio de Janeiro Rio de Janeiro Brazil; ^7^ Centre For Ecology, Evolution and Conservation, School of Environmental Sciences University of East Anglia Norwich UK; ^8^ Center for Biodiversity & Global Change Yale University New Haven Connecticut USA

**Keywords:** dispersal mode, empty forest, functional traits, hunting, plant–animal interaction, tree recruitment

## Abstract

The depletion of tropical frugivorous vertebrates due to overhunting may impair natural forest regeneration. Yet our understanding of how the loss of seed dispersers and browsers along hunting pressure gradients can shift plant community composition and, consequently, trait distributions, is still limited. We assessed the cascading effects of hunting pressure on forest composition by examining species‐level responses and how these are translated into potential shifts in community traits at different life stages. We sampled 4784 trees and 6132 saplings across 30 forest plots along a gradient of hunting pressure in western Brazilian Amazonia, and compiled plant species data on dispersal mode, seed size, wood density, and leaf mass per area (LMA). We tested how hunting pressure affects sapling recruitment probability and sapling‐to‐tree (S:T) abundance ratios based on dispersal syndromes and varying seed sizes. We also evaluated whether hunting influences community‐weighted mean (CWM) wood density, seed size, and LMA of saplings and adult cohorts. Our results show that overhunted forests exhibit significantly lower sapling recruitment probabilities and sapling‐to‐tree (S:T) abundance ratios for large‐seeded endozoochorous species, particularly those bearing seeds larger than 18 mm. In contrast, abiotically dispersed and scatter‐hoarded species exhibited increased recruitment success under high hunting pressure. Hunting pressure had no significant effect on CWMs of wood density, seed size, or LMA for trees and saplings. In our study landscape, up to 249 plant species, encompassing ~⅓ of the species surveyed, may be experiencing seed dispersal limitation and impaired sapling recruitment in heavily hunted forests. Yet, these species‐level responses did not scale up to wholesale changes in community‐wide plant functional composition, potentially due to time‐lag effects. Our study demonstrates that defaunation driven by overhunting triggers early functional shifts in tropical forests by altering plant recruitment patterns, especially for animal‐dispersed species, potentially leading to long‐term changes in forest structure and carbon storage capacity.

## Graphical abstract

We examined how overhunting‐induced defaunation affects the functional composition of tropical tree communities in western Brazilian Amazonia. Using a robust, spatially replicated dataset from 30 forest plots across a well‐defined gradient of hunting pressure, we assessed the impacts of vertebrate depletion on plant recruitment patterns, dispersal syndromes, and key functional traits, including wood density, seed size, and leaf mass per area.

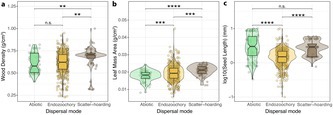

## 1 Introduction

Tropical forests worldwide provide critical ecosystem services for about 1.5 billion people (Vira et al. 2015). These services include climate regulation, carbon storage, and extraction of natural resources such as timber, fruits, oilseeds, plants with therapeutic properties, and bushmeat (Foley et al. 2007; Nunes et al. 2019; Pearce 2001). However, the continuity and magnitude of these services require forest ecosystems hosting a full complement of species and their interactions (Mooney et al. 2009). Overhunting results in a marked decline in large‐bodied vertebrate populations in tropical forests worldwide (Fa and Brown 2009; Harrison et al. 2016; Ripple et al. 2016; Benítez‐López et al. 2019; Ferreiro‐Arias et al. 2025), consequently disrupting important plant–animal interactions (Wilkie et al. 2011). This is particularly the case in the Neotropics (Peres 2000; Peres and Palacios 2007), where key target game vertebrates such as tapir, large primates, peccaries, brocket‐deer, large rodents, and large birds often show clear signs of defaunation (Dirzo et al. 2014; Redford 1992). The loss and declines of these species degrade critical ecosystem services due to their key role in plant community structure through processes such as seed dispersal, density‐dependent seed predation, and seedling browsing (Galetti et al. 2015). Because approximately 80% of all woody plants in Neotropical forests rely on vertebrates for seed dispersal (Howe and Smallwood 1982; Van Roosmalen 1985), disruptions in these mutualistic interactions are expected to significantly alter forest composition and ecosystem functionality (Kurten et al. 2015; Terborgh et al. 2008; Wright, Stoner, et al. 2007).

Overhunting can alter forest composition through population declines in animal‐dispersed large‐seeded plant species, and the associated density compensation of trees and woody lianas dispersed either abiotically or by non‐game vertebrates (Effiom et al. 2013; Harrison et al. 2013; Nunez‐Iturri et al. 2008; Terborgh et al. 2008). Alternatively, forest composition may change through the elevated recruitment success of large‐seeded animal‐dispersed plants if seed predation or seedling herbivory comprises the most affected top–down interactions (Paine and Beck 2007; Poulsen et al. 2013; Roldán and Simonetti 2001; Wright, Hernandéz, and Condit 2007). Selective hunting simultaneously affects prey populations across trophic guilds, whose interactions could be disrupted depending on the balance of prevalent agents that may either decline or increase indirectly due to overhunting (Gardner et al. 2019; Stoner et al. 2007; Wright et al. 2000). Consequently, predicting the directional pathways of compositional changes in overhunted tropical forests is far from straightforward (Beckman and Muller‐Landau 2007; Kurten et al. 2015), rendering plant recruitment outcomes counterintuitive at even the best‐studied forest sites (e.g., Hazelwood et al. 2020).

Regardless of possible divergences in forest composition responses to complete or partial large‐vertebrate defaunation, the depletion of seed dispersers can result in greater changes in forest functional composition compared to the depletion of seed predators and herbivores (Kurten et al. 2015). Declines in the abundance of plant species dispersed by large‐bodied frugivores and their replacement by those dispersed abiotically or by small‐bodied frugivores are further expected to favor plant traits associated with the latter in community‐wide transitions in functional trait composition (Kurten et al. 2015; Vaessen et al. 2023). Plant species potentially succumbing to dispersal limitation include primarily large‐seeded, heavy‐wooded, and higher‐statured trees, which characterize the slow‐growing, carbon‐dense flora with conservative biomass accumulation strategies (Bello et al. 2015; Peres et al. 2016). In addition to dispersal limitation, overhunting often decimates populations of large herbivores, reducing top‐down browsing pressure on saplings and potentially altering selective pressures on leaf and wood traits (Vaessen et al. 2023). Under this scenario, one might expect an increase in species with more palatable leaves and tissues (i.e., lower leaf mass per area [LMA] and wood density [WD]) if herbivory is a key force shaping plant communities (Poorter et al. 2004; Vaessen et al. 2023). Therefore, the gradual floristic replacement of conservative species (slow‐growing, heavy‐wooded) by acquisitive strategies (fast‐growing, light‐wooded species) can downgrade forest carbon storage to varying extents, as shown in large‐scale modeling studies (Bello et al. 2015; Peres et al. 2016; Osuri et al. 2016). However, functional profiles of plant communities are not only affected by species identity and performance but also by climatic and edaphic variables (Joswig et al. 2022).

Despite the growing number of studies examining the cascading effects of defaunation on tree species composition (Effiom et al. 2013; Holbrook and Loiselle 2009; Harrison et al. 2013; Kurten et al. 2015; Nunez‐Iturri et al. 2008; Terborgh et al. 2008), identifying clear patterns of functional responses has proven elusive. Defaunation effects on tree regeneration are often investigated during only the early stages of seedling recruitment (Effiom et al. 2013; Holbrook and Loiselle 2009; Vanthomme et al. 2010), which implicitly assume that changes at this (st)age class persist into subsequent cohorts, thereby defining the composition and diversity of future tree communities. However, the high density‐dependent mortality of seedlings near parent trees (i.e., Janzen‐Connell effects) may not be reflected at later stages of tree ontogeny (Beck et al. 2013; Theimer et al. 2011). As such, long‐distance dispersal limitation can increase the spatial clustering of species that rely on potentially overhunted dispersal agents, rather than promoting high sapling mortality (Bagchi et al. 2018; Lamperty et al. 2025). Furthermore, a common limitation is that studies often contrast only the extremes of defaunation, either by using experimental approaches that fully exclude large vertebrates (Camargo‐Sanabria et al. 2015; Granados et al. 2018), or by comparing pristine versus heavily overhunted sites (e.g., Terborgh et al. 2008), thus overlooking the vast and ecologically critical ‘half‐empty’ forests under intermediate hunting pressure (HP) that now dominate much of the tropics (Benítez‐López et al. 2019; Bogoni et al. 2020; Ferreiro‐Arias et al. 2025; Harrison et al. 2016).

Here, we investigate the cascading effects of defaunation on the functional tree composition of Amazonian forests throughout a marked gradient of HP. Specifically, we aim to (1) assess whether HP alters sapling recruitment for tree species with different dispersal modes and varying seed sizes, and (2) evaluate if these impacts scale to the community level, shifting forest functional composition and lowering community means of traits positively related to seed size. We use a robustly replicated design over a large meta‐landscape across structurally intact forest sites ranging from no or very low HP to persistently overhunted sites. This gradient drives a marked alteration in vertebrate community composition, characterized by large declines in the biomass of frugivores and grazers and a ~2.7‐fold downsizing in the assemblage‐wide vertebrate size structure from the least to the most hunted sites (Scabin and Peres 2021).

Based on a comparative approach using quantitative inventories of two tree (st)age classes (saplings and adult stems) along this hunting‐induced defaunation gradient, we evaluated the cascading effects of HP on forest composition by assessing species‐level responses and how these are translated into potential shifts in community traits at different life stages. We expected that the probability of sapling recruitment and the abundance of saplings that are dispersed abiotically or by scatter‐hoarding would increase in heavily hunted sites at the expense of endozoochorous species. Further, we also expected that, among endozoochorous species, the abundance of saplings of large‐seeded species would decrease with increasing HP. Additionally, we hypothesized that directional changes in sapling composition would promote a concomitant alteration in community‐wide patterns of WD, seed size, and leaf economics. These key functional traits are fundamental axes of plant strategy while being directly or indirectly affected by hunting due to their correlations with seed size (Galetti et al. 2013; Peres et al. 2016; Bello et al. 2015; Vaessen et al. 2023). Seed size reflects dispersal limitation and the loss of large frugivores (Galetti et al. 2013; Peres et al. 2016); WD positively correlates with seed size while being a determinant of carbon storage and growth rates (Bello et al. 2015; Peres et al. 2016); and LMA indicates investment in leaf structure and potential response to released herbivory pressure (Vaessen et al. 2023). We expected the community‐weighted mean seed size and WD to be reduced due to the lack or reduction of recruitment by trees dispersed primarily by large frugivorous vertebrates, particularly lowland tapir and large primates which have experienced severe local declines in our hunted and overhunted study sites (see Scabin and Peres 2021). In addition, we expected the community weighted mean of LMA to be higher in heavily hunted sites, as a response to ecological release from browsing pressure by large‐bodied herbivore declines.

## 2 Methods

### 2.1 Study Area

**FIGURE 1:**
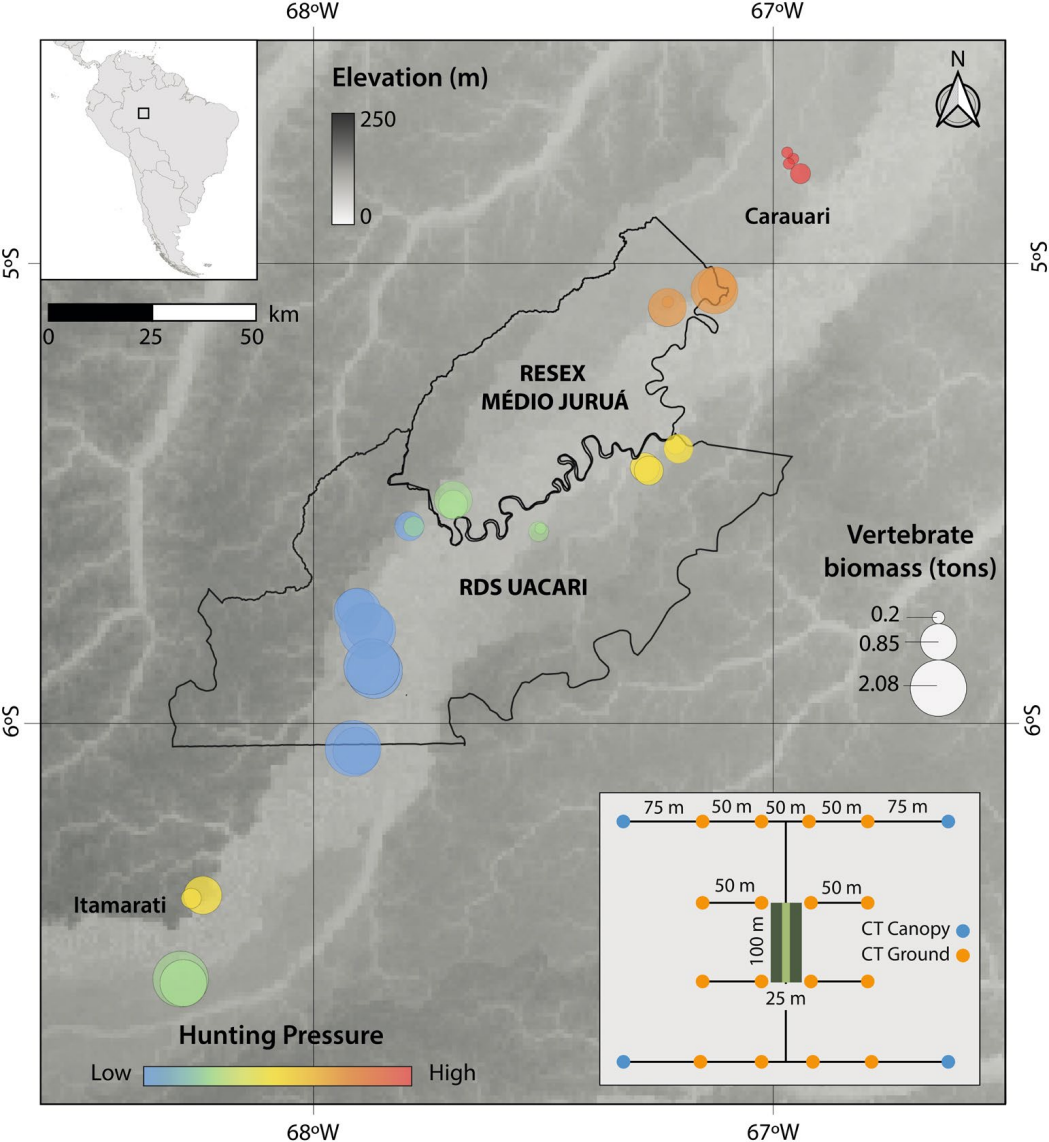
Geographic location of the Médio Juruá study area in western Brazilian Amazonia. The RESEX Médio Juruá and RDS Uacari reserve boundaries are indicated in black. Circles indicate the location of our 30 permanent tree and sapling plots and are color‐coded according to our proxy of hunting pressure and circle size denotes the estimated amount of vertebrate biomass per study site (see Scabin and Peres 2021). The background map represents elevation above sea level in meters. The panel on the bottom right depicts a schematic drawing of the camera trap (CT) and vegetation plot sampling design. Orange and blue circles denote understorey and canopy camera traps, respectively, used for vertebrate surveys (see Scabin and Peres 2021). The central dark green rectangle represents a 0.25‐ha permanent adult tree plot (100 × 25 m), while the light green rectangle indicates a nested 0.05‐ha (100 × 5 m) permanent sapling plot.

This study took place in the Médio Juruá region of western Brazilian Amazonia (Figure 1), including two large contiguous sustainable use protected areas and adjacent landscapes containing two urban centers. This roughly represents the ~1200‐km middle‐third section of the Juruá River, the second‐longest white‐water tributary of the Amazon River. The two protected areas include the 253,227‐ha Medio Juruá Extractive Reserve (RESEX Médio Juruá; 5°33′54″ S, 67°42′47″ W), created in 1997 and legally occupied by ~2000 people distributed across 15 villages; and the 632,949‐ha Uacari Sustainable Development Reserve (RDS Uacari; 5°43′58″ S, 67°46′53″ W) created in 2005, and occupied by ~1200 people across 32 villages (IBGE–Instituto Brasileiro de Geografia e Estatística 2018). The nearest towns are Carauari (28,076 residents) and Itamarati (7888 residents), which are located 88 and 120 km downstream and upstream from these reserves, respectively (Figure 1; IBGE–Instituto Brasileiro de Geografia e Estatística 2018). The Médio Juruá region experiences a wet tropical climate with a mean annual temperature of 27.1°C and a mean annual rainfall of 3679 mm, with the wettest period between November and April. Two different forest types comprise the study landscape: seasonally flooded (*várzea*) forests, which account for ~20% of the study region, characterized by enriched Andean alluvial soils and lower floristic diversity, and the dominant (~80%) unflooded forest (*terra firme*), which exhibits higher floristic diversity and comparatively lower soil fertility (Hawes and Peres 2016). Our study took place in unflooded forest sites on old *paleovárzea* sediments, hereafter referred to as *terra firme* forests for simplicity, although we recognize that these forests may diverge in their floristic macromosaics from *terra firme* forests farther upland (Assis et al. 2015). Our vegetation plots were established along forest sites that had experienced or currently experience either subsistence or commercial hunting to varying degrees but a very limited history of selective logging and no wildfires. The introduction of modern hunters wielding fireweapons dates back to the first stage of occupation by the first rubber tappers in the Médio Juruá region during the early days of the rubber boom in the late 19th century (~1870–1890), following the arrival of the rubber extractive industry (Derickx and Transferetti 1992; da Silva Guimarães et al. 2022).

### 2.2 Hunting Pressure

We built a proxy of HP for each plot based on the geographic distance to and size of regional‐scale human settlements, including all isolated households, villages, and the towns of Carauari and Itamarati (see details in Scabin and Peres 2021). This proxy is supported by previous studies in the same study areas (Abrahams et al. 2017; Nichols et al. 2013; Scabin and Peres 2021) and was calculated using the following equation:

$$\mathrm{HP}=\sum_{i}^{n}\frac{S\left(\mathrm{vil}\right)}{\sqrt{d\left(\mathrm{vil}\right)}}+\frac{S\left(\mathrm{caf}\right)}{\sqrt{d\left(\mathrm{caf}\right)}}+\frac{S\left(\mathrm{caf}\right)}{\sqrt{d\left(\mathrm{caf}\right)}}$$

where *S* represents the human population of a given settlement and *d* represents the distance from the plot centroid to that settlement. The subscripts vil, caf, and ita denote the nearest village and the towns of Carauati and Itamarati, respectively. We measured the Euclidean distance from the centroid of each vegetation plot to the nearest community and the nonlinear dry‐season navigation (fluvial) distance to the towns using ArcGIS10.3. Human population size was based on data sourced from Brazil's National Census Bureau (IBGE–Instituto Brasileiro de Geografia e Estatística 2018), Instituto Juruá (IJ) and Fundação Amazônia Sustentável (FAS).

### 2.3 Study Design

A total of 15 forest sites and 30 vegetation plots were studied along the entire ~500 km long spectrum of HP throughout the Juruá study landscape. From May to December 2017, we established at each forest site (1) two 0.25‐ha plots (25 × 100 m), at least 1 km apart, where all trees and arborescent palms ≥ 10 cm diameter at breast height, DBH (hereafter, trees) were surveyed; and (2) two 0.05‐ha (5 × 100 m) sapling subplots nested within the larger tree plots, where all juvenile trees and arborescent palms ≥ 1 m in height and with a stem diameter of 1–5 cm at the point of measurement (hereafter, saplings) were surveyed (Figure 1). Vegetation plots were placed at the centre of camera‐trap grids aimed at estimating arboreal and ground‐dwelling vertebrates (Scabin and Peres 2021). Our camera trap (CT) surveys conducted concurrently at the same plots have shown that large‐bodied frugivores and grazers are either absent or occur at very low densities in the peri‐urban (< 30 km) forests near Carauari and Itamarati (see Scabin and Peres (2021) for further details).

All living plant stems were recorded if the base of their trunk was located entirely or partially within the plot boundaries, regardless of whether their crown extended outside the plot. All living individuals were measured and number‐tagged at 1.3 m height for trees and 1 m for saplings. The floristic inventory was conducted in July 2018, when all species were identified in the field to the finest possible taxonomic level. Species identification was carried out by a professional parabotanist with over 30 years of field and herbarium experience at the Brazilian National Institute for Amazonian Research (INPA), Manaus, Brazil. Voucher specimens of all individuals that could not be identified in the field were collected and subsequently identified at the INPA herbarium with the assistance of another parabotanist and then deposited at the EAFM herbarium of the Instituto Federal de Educação, Ciência e Tecnologia do Amazonas registered at the numbers 18.5191 to 18.690 and 18.910 to 19.027 (IFAM, Manaus). Considering all individuals sampled, 99% were identified at the species level, 0.9% at the genus level, and 0.1% (8 individuals) remained unidentified. We used the Taxonstand 22.2 R package (Cayuela et al. 2012) in R v.3.5.3 (R Core Team 2024) to harmonize plant nomenclature and correct for synonyms based on The Plant List (http://www.theplantlist.org).

### 2.4 Sapling Recruitment

We examined changes in plot‐scale species‐specific density (D) between trees and conspecific saplings at each site based on the log‐transformed sapling‐to‐tree ratio of abundances (S:T) considering all stems (trees and palms) within each plot and subplot, a metric used elsewhere (Hazelwood et al. 2020; Jones et al. 2019; Terborgh et al. 2008). This abundance log‐ratio was calculated for every species that had been recorded at least once both as a tree and a sapling in each plot, following the equation:

$$S:T=\log_{10}\;\left[\left(D_{\text{sapling}\left(i\right)}\right)/\left(D_{\text{tree}\left(i\right)}\right)\right]$$

Sapling subplots spanned the entire length of each tree plot and were therefore representative of the sapling layer underneath all trees within each plot. S:T values were thus used as a proxy of plant recruitment so that positive values (> 0) indicate a greater density of saplings compared to co‐occurring conspecific trees, while negative values (< 0) indicate lower density of saplings compared to conspecific trees. Because we had instances where species had been recorded as adult trees, but not at the sapling stage, we also coded each species as 0 or 1 if absent or present in a plot.

### 2.5 Plant Functional Traits

We directly measured or assigned values or categories based on the literature for plant functional traits that are widely recognized to be critical for tree recruitment (Westoby 1998), which we expected to be indirectly affected by long‐term seed dispersal limitation induced by overhunting. These include three continuous (WD, LMA, and seed size [seed length—SL, seed width—SW, and seed mass—SM]), and one categorical trait (seed dispersal mode).

#### 2.5.1 Wood Density

To obtain species‐specific WD, we used a 5.15 mm Haglöf increment borer to sample three wood cores from different individuals for each of the 250 most abundant tree species, amounting to a total of 700 wood core samples. Wood cores were extracted perpendicularly to the bark at about 1 m height from trees > 10 cm DBH. To encompass the variation in heartwood‐to‐bark WD, cylindrical samples were equivalent to the approximate length of the bole radius (DBH × 0.5). Each wood core was first wrapped in filter paper and stored in a box containing silica gel for initial drying to avoid fungal attack for 5 days and then stored into labeled plastic straws. We then rehydrated all wood samples for 24 h at the Plant Functional Laboratory at Instituto Nacional de Pesquisas da Amazônia (INPA). We subsequently obtained the green volume of each core through the water displacement method, using a beaker of water placed on a digital balance (precision ≈0.01 g) which was re‐zeroed each time. All wood cores were then oven‐dried at 105°C for 72 h, at which point they were dry‐weighted. WD values were calculated by dividing the dry wood core weight by the green volume of each sample (Chave 2002). As we used the water displacement method, we obtained the wood specific gravity (WSG) value, defined as the relationship between the dry wood mass and the wood volume at saturation point in relation to the volume of water; for simplicity, this is hereafter referred to as WD. To obtain species‐specific WD values, we averaged the WD values across all samples of the same species. WD values were obtained for 54.8% of all tree stems surveyed. For stems lacking WD values obtained from our own in situ destructive sampling, we used data from the Global Wood Density Database (Chave et al. 2009; Zanne et al. 2009) including only values from South America. In total, 57.23% of all individuals were assigned to a WD value at the species level, 21.54% at the genus level, and 21.09% at the family level, while 0.13% (17 stems) had no information on WD available (Table S1).

#### 2.5.2 Leaf Mass Per Area

LMA is a plant functional trait related to the energy investment into leaf production, so that higher LMA values are associated with greater energy investment into long‐lived leaves, while lower values are related to short‐lived leaves (Poorter 2007). We collected 2327 leaves belonging to 477 tree species within all subplots. All leaves were manually collected at random in the understorey according to access to terminal foliage, prioritizing foliage showing healthy condition. We collected one leaf per sapling. Each leaf was number‐tagged and had a 4‐cm^2^ section cut using a shape cutter. All samples were placed in paper envelopes and oven‐dried at 65°C for 48 h and then weighed on a digital scale (precision ≈0.001 g). The leaf section weight divided by 2 cm corresponds to LMA (expressed as g•cm^−2^). Species‐specific LMA was obtained for 63.9% of all stems surveyed. For all individuals for which species‐level data were lacking, we imputed data using the mean genus‐level (10.8% of all stems) or, if not available, the mean family‐level value (2.9% of all stems, Table S1).

#### 2.5.3 Seed Dispersal Mode and Seed Size

Seed dispersal mode for all woody tree species was retrieved from published literature and field guides on the Amazonian flora (Amaral et al. 2009; Baraloto and Forget 2007; Cornejo and Janovec 2010; Hammond and Brown 1995; Van Roosmalen 1985). Functional groups related to seed dispersal mode were defined as: (1) abiotic (wind, water, and ballistic), (2) scatter‐hoarded by large rodents, and (3) endozoochory. While ballistic dispersal (autochory) is ultimately powered by the plant's own tissues, we assigned it here to the abiotic dispersal syndromes for consistency across major categories, as it does not involve an animal dispersal vector. We also compiled data on SL, SW, and dry SM by species and/or genus, which was supplemented by data available online from the Kew Seed Information database (Kew (Royal Botanic Gardens) 2008). Seed diameter (width) is usually employed as an index of seed size because most frugivores swallow the seeds lengthwise. However, we used SL in our analyses because this metric was available for a larger number of species at either species or genus level (Table S1; see also Vanthomme et al. 2010); and because it was highly correlated with SW and SM, particularly for endozoochorous species (Figure S1). We also used a 12‐mm threshold of SW (Galetti et al. 2013; Bello et al. 2015) and 18 mm SL to determine whether seeds could potentially incur dispersal limitation (Figure S1). This value is slightly higher than the mean SL across endozoochorous species in our dataset.

### 2.6 Environmental Covariates

Considering that climatic and edaphic variables such as water and soil‐nutrient availability can predict the functional composition of forests (Fortunel et al. 2014), we obtained data on soil fertility and water availability for each of the 30 plots. At each plot, we collected three soil samples of ~5 g each using a soil auger with samples extracted near the plot centroid, at least 10 m apart. Soil samples were air‐dried and then sealed in plastic bags before analysis at the INPA Soil Chemistry Laboratory. Soil fertility was represented by the cation exchange capacity (CEC) as the sum of Ca^+2^, Mg^+2^, and K^+^ concentrations measured as cmol kg^−1^. We used the vertical distance to the nearest drainage (VDND) as a proxy of soil water availability, as it has been shown to exert a large influence on vegetation composition across Amazonian terra‐firme forests (Schietti et al. 2014). VDND measures the elevation difference to the closest stream along the topographic flow path of the nearest water sources, providing a proxy for local water availability and drainage for plants. For each plot, we calculated VDND using the Height Above the Nearest Drainage (HAND) algorithm proposed by Rennó et al. (2008) based on the 30‐m resolution Digital Elevation Model (DEM) topography available from the Shuttle Radar Topography Mission (SRTM). The vertical distance grids generated by the HAND algorithm are available from the Brazilian Space Agency (INPE) website (www.dpi.inpe.br).

### 2.7 Data Analysis

We build a set of (generalized) linear mixed models (LMMs) to explore the impacts of hunting on forest composition at both species and community levels. Before modeling, we calculated Pearson correlation coefficients (*r*) to test for multicollinearity among explanatory variables at the plot level, where *r* > |0.7| indicates high multicollinearity (Zuur et al. 2010). All variables were largely uncorrelated and therefore retained in all models (Figure S2). We further examined the phylogenetic signal (*λ*) in the residuals of all models by constructing a phylogenetic tree using our species list and GBOTB extended TPL tree using the “*V.PhyloMaker2*” package (Jin and Qian 2022). Subsequently, we calculated Pagel's *λ* using the *phytools* package (Revell 2012). We further calculated the conditional and marginal *R* ^2^ values for all mixed models using the function *r2_nakagawa*() from the *performance* package (Lüdecke et al. 2021).

First, to determine to what degree HP affects the probability of sapling recruitment for plant species with different dispersal modes (i.e., species‐level responses), we fitted a Generalized Linear Mixed Model (GLMM) with a binomial family. To this end, we used as a response variable the presence (1) or absence (0) of at least one sapling of a focal species in a plot, given the presence of a conspecific adult tree in that same plot. We used species identity as a random effect to account for the non‐independence of multiple observations from the same species and as fixed effects the interaction between HP and dispersal mode (abiotic vs. scatter‐hoarded vs. endozoochory), while controlling for potential confounding factors with additive effects of CEC, VDND, and the number of conspecific adult trees in the plot.

Second, we tested the effect of HP on the abundance of saplings for plant species with different dispersal modes using a LMM. We used conspecific sapling‐to‐tree abundance ratios (S:T) as the response variable while retaining the same fixed and random effect structures employed in the aforementioned GLMM.

Third, we tested the hypothesis that HP will impact more negatively larger‐seeded species compared to small‐seeded species by fitting a LMM using only a subset of data for endozoochorous species. We used as a response variable the conspecific sapling‐to‐tree abundance ratios (S:T) and as fixed effects the interaction between SL and HP together with the additive effects of CEC and VDND. We used SL as a trait metric related to seed size instead of SW or SM due to its broader taxonomic coverage at the species level in our dataset and tested the correlation between SL, SW, and SM (Table S1, Figure S1). We further included a nested random effect of species identity within plant family since a single random intercept for species identity did not account for the phylogenetic signal in the model residuals (*λ* = 0.43; LogLikelihood = −15.99; *p* value = 0.012).

Finally, to examine the relationship between continuous functional traits (i.e., community‐level responses), we first investigated the Pearson correlation structure between WD, LMA, and SM for all plant species, grouping them according to their dispersal mode. We then examined to what degree these functional traits differed across seed dispersal modes using pairwise *t* tests. To understand whether assemblage‐wide distributions in continuous traits were affected by HP, we used the Community Weighted Mean (CWM) approach in which traits were scaled by individual contributions to community‐level aboveground biomass (Muscarella and Uriarte 2016). We calculated community means (CWM) weighted by the basal area (BA) of individual stems (*i*) using the following equations:

$${\mathrm{BA}}_{i}=\pi \cdot {\left(\frac{{\mathrm{DBH}}_{i}}{2}\right)}^{2}$$

where DBH is the diameter at breast height of the stem *i*; and:

$${\mathrm{CWM}}_{j}=\frac{\sum_{i=1}^{N}\left({\mathrm{BA}}_{\mathrm{ji}}\cdot T_{i}\right)}{\sum_{i=1}^{N}{\mathrm{BA}}_{\mathrm{ji}}\;}$$

where *T* is the trait (i.e., WD, leaf mass area or SL), *j* is the plot, and *i* refers to individual stems. To assess community‐level responses of different trait values in different cohorts (i.e., adult trees vs. saplings), we fitted three different linear models using the CWMs of WD, LMA, and SL as response variables but the same fixed effect structure consisting of the interaction between HP and life stage (adult vs. sapling) and the additive effects of VDND and CEC. To this end, we only used values for plant species with trait values available at the species level or imputed at genus level. All analyses were conducted in R v.3.5.3 (R Core Team 2024).

## 3 Results

### 3.1 Plot‐Scale Variables and Vegetation Inventories

We recorded a total of 4784 trees across the 30 permanent tree plots (total area = 7.5 ha) and 6132 saplings across the 30 sapling subplots (1.5 ha), which represented 846 species, 268 genera, and 67 families (Table S2). Seed dispersal mode of over 68.93% of all trees and 80.86% of all saplings, which encompassed 79.1% of plant species found in all surveyed plots, was assigned to endozoochory (Table S3). Abiotically dispersed trees represented 8.68% of trees and 6.31% of saplings and 12.1% of all species, whereas species scatter‐hoarded by large rodents represented 22.4% of all trees, 13.3% of all saplings, and 8.8% of all plant species (Table S3). The mean VDND across plots was 16.3 m (range: 0–53.6 m). Soil CEC averaged 0.80 cmol/kg (range: 0.07–4.87 cmol/kg). The mean HP index was 4.6 (range: 2.4–13.8).

### 3.2 Within‐Plot Community Drift Responses to HP

**FIGURE 2:**
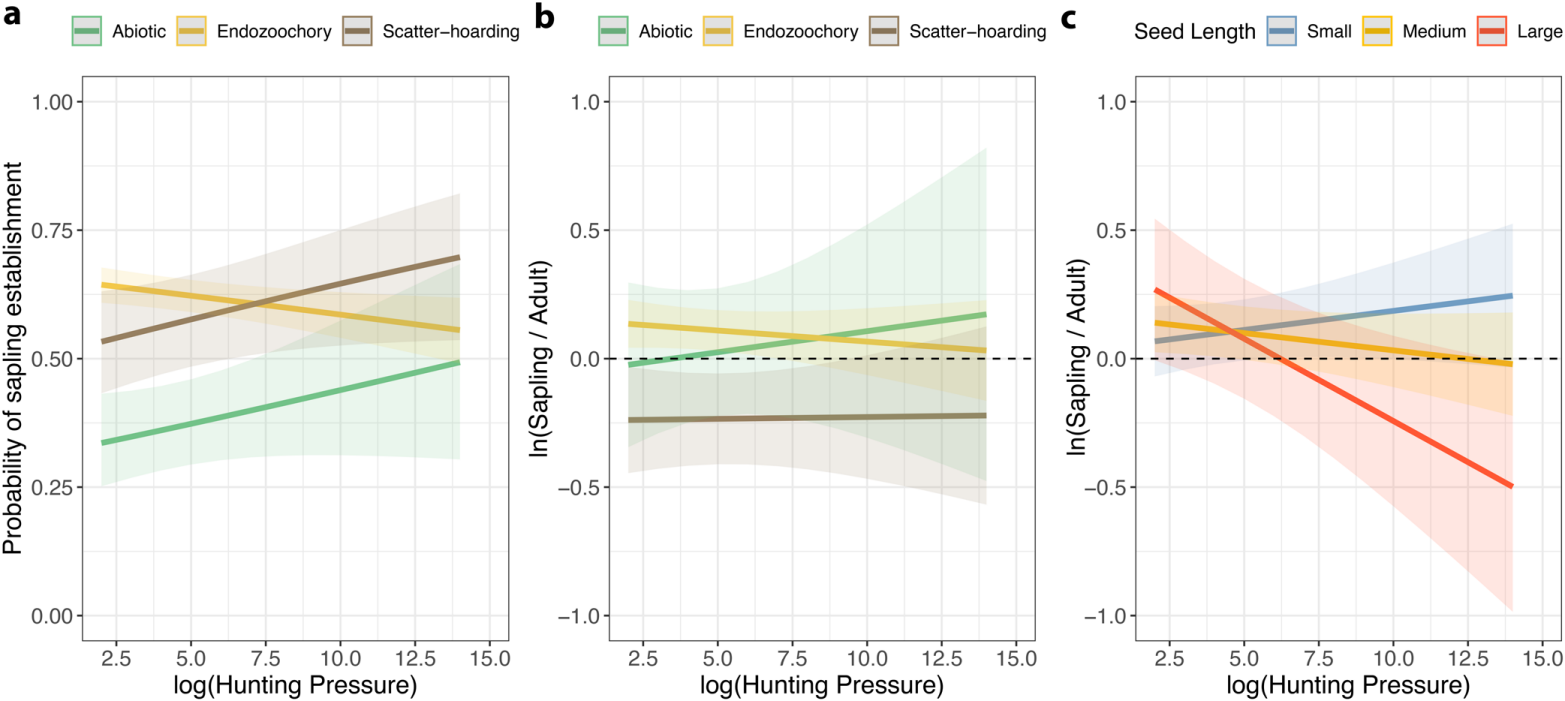
Marginal effects from (G)LMMs showing the effects of hunting on (a) sapling establishment probability (left panel) and (b) S:T ratios (central panel) of abiotically‐dispersed (green), endozoochorous (yellow), and scatter‐hoarded (brown) plant species. The right panel (c) shows S:T ratios for endozoochorous plant species across different size classes: Small‐seeded (1 mm < 1st quantile), medium‐sized (18 mm, equivalent to a seed width threshold of 12 mm), and large‐seeded (50 mm > 3rd quantile).

**FIGURE 3:**
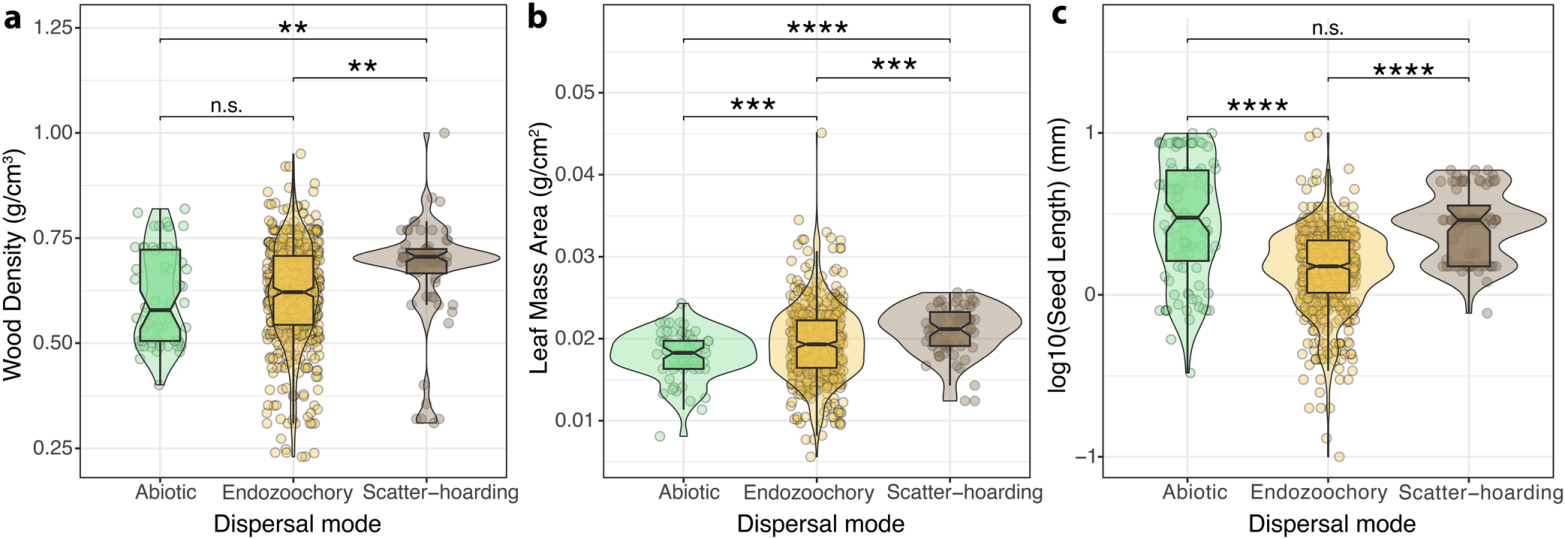
Boxplots showing mean functional trait values for (a) wood density, (b) leaf mass per area (LMA), and (c) seed length for plant species with different seed dispersal modes (abiotic, endozoochory, scatter‐hoarded). Pairwise *t*‐test and *p*‐values are indicated by the following significance levels: **** for *p* ≤ 0.0001, *** for 0.0001 < *p* ≤ 0.001, ** for 0.001 < *p* ≤ 0.01, * for 0.01 < *p* ≤ 0.05, and n.s. for *p* > 0.05.

We found no evidence of a phylogenetic signal in the residuals of mixed‐effects models testing the effects of HP on sapling recruitment probabilities (*λ* = 0.3; *p* = 0.068), on S:T abundance ratios for all species (*λ* = 0.26; *p* = 0.173), and on S:T abundance ratios for endozoochorous species (*λ* = 0.00007; *p* = 1). Overall, the probability of sapling recruitment was significantly affected by dispersal mode, positively related to VDND, and negatively related to the number of conspecific adult trees (Table S4). Further, the probability of sapling recruitment for endozoochorous plant species significantly decreased as HP increased (Figure 2a). Conversely, sapling recruitment probability for abiotically dispersed and scatter‐hoarded species increased at heavily hunted sites (Figure 2a). However, we did not find any effect of HP on the S:T abundance ratio for species with different dispersal modes, nor an effect of soil fertility or elevation above the water table (Figure 3b, Table S5). However, S:T abundance ratios for endozoochorous plant species were significantly influenced by HP for species bearing variable‐sized seeds (Figure 2c, Table S6). Saplings of small‐seeded endozoochorous plant species (i.e., SL ≤ 1 mm) increased in abundance with HP, while large‐seeded trees (i.e., > 18 mm in length and > 12 mm in width) showed negative S:T abundance ratios. Based on this seed size threshold (Figure S1), 249 endozoochorous plant species (~29%) across all plots showed signs of seed dispersal limitation and impaired sapling recruitment in heavily hunted forests (Figure 2c).

### 3.3 Functional Traits Across Dispersal Modes

We found that species dispersed by large scatter‐hoarding rodents exhibited statistically higher WD values compared to endozoochorous and abiotic‐dispersed plant species (Figure 3a). No differences in WD were found between endozoochorous and abiotically dispersed plant species. We found statistically significant differences in LMA between all dispersal modes, with scatter‐hoarded trees showing the highest values, followed by endozoochorous and abiotically dispersed species (Figure 3b). Seed size in plant species with abiotic and scatter‐hoarding dispersal modes was larger than that in endozoochorous species (Figure 3c).

For all species surveyed, LMA was positively correlated with WD (*r* = 0.23, *p* < 0.001) and seed size (*r* = 0.16, *p* < 0.001), but WD and seed size were uncorrelated (*r* = 0.065, *p* = 0.084, Figure S3a). Trait correlations displayed different patterns among the three major dispersal modes (Figure S3b). All traits were positively and statistically significantly correlated for endozoochorous plant species (Figure S3b). In turn, WD and seed size were negatively correlated for abiotically dispersed seeds (*r* = −0.45, *p* < 0.001), whereas traits in scatter‐hoarded plant species were uncorrelated (Figure S3b).

### 3.4 Community‐Wide Functional Responses to HP

**FIGURE 4:**
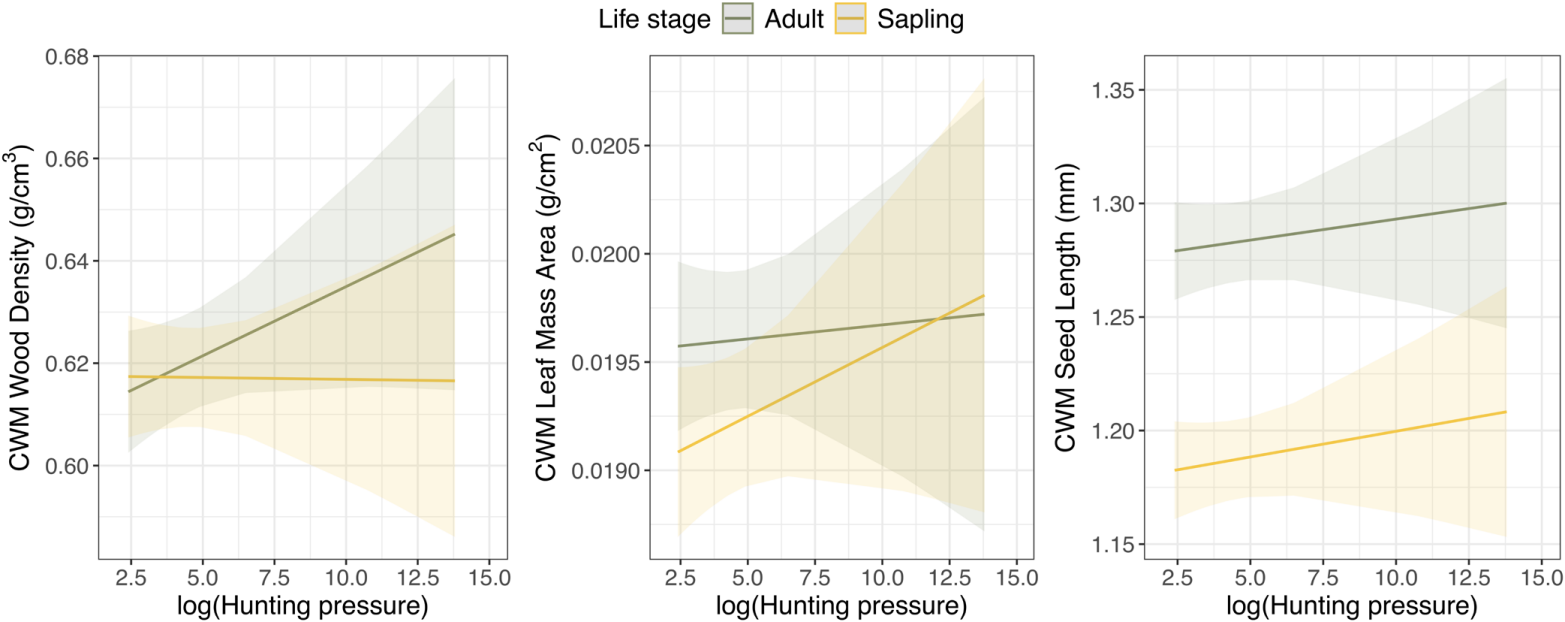
Overall nonsignificant relationships between community‐weighted mean (CWM) traits, including wood density, leaf mass per area and seed size, and degree of hunting pressure for both trees (gray lines) and saplings (yellow lines) across all forest plots. Solid lines and shading represent linear fits and 95% confidence interval regions.

Linear models showed that community weighted mean WD, LMA, and SL for both trees and saplings were unrelated to our metric of HP or other environmental variables (Tables S7–S9). Differences between CWM trait values between saplings and adults were unapparent, except for CWM seed size, which was consistently greater in adults compared to saplings along the full HP gradient (Figure 4, Table S9). Although plots exposed to heavier HP on average contained slightly higher densities of heavy‐wooded trees, this trend was no longer visible for saplings as these differences were not statistically significant (Figure 4, Table S7).

## 4 Discussion

This study uses a robustly replicated landscape‐scale design to examine the degree to which functional profiles of tree assemblages in high‐diversity Amazonian forests are affected by their history of modern HP dating back to the heyday of the rubber boom (Derickx and Transferetti 1992; da Silva Guimarães et al. 2022). Forest plots subjected to heavy HP showed a decline in the recruitment of endozoochorous tree species, whose probability of sapling establishment was lower than that of trees dispersed by abiotic agents or scatter‐hoarding rodents. Our findings also show signs of the disruption of seed dispersal mutualisms in heavily hunted forest, with large‐seeded endozoochorous tree species experiencing lower recruitment than those dispersed by small‐to‐medium‐sized frugivores that are either ignored by hunters or are insensitive to hunting. Mean values across surveyed tree species for WD, LMA, and seed size were significantly different across major seed dispersal modes, except for trees dispersed by large scatter‐hoarding rodents and abiotic agents, which showed similar SL values, and between abiotic and endozoochorous species in terms of WD. Furthermore, community‐wide patterns of continuous plant functional traits were not significantly different across the entire HP gradient along a ~500 km longitudinal section of the Juruá River.

### 4.1 Seed Size and Seed Dispersal Mode

Peri‐urban areas along the Juruá River have experienced the longest and most concentrated hunting footprint since the height of the rubber boom (Abrahams et al. 2017; Scabin and Peres 2021). Yet, they still represent continuous tracts of otherwise relatively undisturbed forest that are not entirely depleted of large‐bodied vertebrates, likely because source‐sink dynamics with surrounding nonhunted areas have been maintained (Abrahams et al. 2017, Scabin and Peres 2021). Nevertheless, even in light of these mitigating factors that potentially buffer the effects of hunting, it was possible to detect non‐random directional shifts in the compositional profile of plant functional groups, which are consistent with previous studies in the Amazon (Nunez‐Iturri et al. 2008; Terborgh et al. 2008), Mesoamerica (Kurten et al. 2015; Wright, Stoner, et al. 2007), South‐East Asia (Harrison et al. 2013), and Afrotropical forests (Effiom et al. 2013; Vanthomme et al. 2010). The overall effect size of intermediate to high HP on forest composition that we observed appears to be less pronounced than in more binary studies that compared persistently overhunted forests and adjacent nonhunted protected areas (Nunez‐Iturri et al. 2008; Terborgh et al. 2008). However, we still found evidence of limited recruitment in endozoochorous plant species in the most hunted sites, particularly those bearing large seeds. This calls for a greater focus on better replicated studies including “half‐empty forests” (*sensu* Redford and Feinsinger 2001) that may allow us to identify vertebrate biomass thresholds at which tropical ecosystems could shift towards communities dominated by abiotic, small‐seeded, and/or scatter‐hoarded plant species dispersed by vertebrate species that are less sensitive to persistent HP. Here, we provide evidence that this apparently takes place at intermediate levels of decay in vertebrate biomass, as our findings can be simultaneously contextualized within the description of the regional hunting‐induced gradient of vertebrate biomass (see Scabin and Peres 2021).

At heavily hunted Juruá sites, in addition to declines in large‐bodied frugivore biomass, white‐lipped peccaries ( *Pecari tajacu* ) were also recorded at much lower densities (Scabin and Peres 2021). The low abundance of large‐bodied seed predators often facilitates seedling recruitment for some large‐seeded species (Silman et al. 2003; Wright et al. 2000). On the other hand, echimyid rodent populations such as spiny rats (*Proechimys* spp.) and the seed predation pressure they exert can benefit from large mammal declines through density compensation mechanisms (Galetti et al. 2015), as has been reported in the study region (Scabin and Peres 2021). Accordingly, the overall quantitative balance between mutualistic and antagonistic plant–animal interactions, such as seed dispersal, seed predation, and herbivory, likely defines both the direction and magnitude of defaunation effects on tree regeneration (Gardner et al. 2019). Consequently, it is important to further dissect which of these interactions may have been either facilitated or penalized to understand the effects of partial defaunation on the floristic composition of tropical forests given their residual assemblages of vertebrate frugivores.

Several studies reporting forest compositional changes due to defaunation argue that seed size is a good predictor of the strength of trophic cascades on tree regeneration (Dirzo et al. 2007; Wright, Stoner, et al. 2007; Galetti et al. 2013; Bello et al. 2015; Peres et al. 2016). Our results further substantiate these patterns by signaling reduced sapling abundance compared to co‐occurring adult conspecific trees for large‐seeded plant species. Despite the generally strong association between seed size and their seed‐dispersal vectors, given the physical constraints of ingestion and gut passage, some non‐game species such as frugivorous bats (e.g., *Artibeus* spp.) can also disperse large seeds (Melo et al. 2009). In addition, large scatter‐hoarders such as agoutis (*Dasyprocta* spp.) and acouchis (*Myoprocta* spp.) are largely harvest‐insensitive due to their high reproductive rates and may boost the survival and recruitment of some large‐seeded trees through active primary or secondary dispersal (Galetti et al. 2010). However, evidence for these compensatory mechanisms was weak or even non‐existent in our heavily hunted sites, precluding the notion of dispersal redundancy (Culot et al. 2017). Although our findings indicate reduced recruitment of large‐seeded plant species in heavily defaunated sites, this did not translate into community‐level changes in mean seed size or a radical shift towards plant communities dominated by abiotically dispersed or synzoochorous species. This lack of effects could be attributed to the modern history of HP in the Médio Juruá region dating from ~1870–1890 (Abrahams et al. 2017; Scabin and Peres 2021; da Silva Guimarães et al. 2022) and the long generation time of many Amazonian tree species with long‐lived trees reaching > 1000 years (Chambers et al. 1998; Laurance, Nascimento, et al. 2004). For species reliant on large‐bodied, harvest‐sensitive primates (e.g., *Ateles* spp., *Lagothrix* spp.), the ecological consequences of dispersal collapse may not yet be fully visible in our study region (Peres et al. 2016). This can be particularly true for families like Sapotaceae, whose species showed signs of dispersal limitation at our study sites but are characterized by extremely long lifespans (Laurance, Oliveira, et al. 2004). For example, key species in our study region exhibit longevities of centuries such as *Pouteria guianensis* (720 years), *Manilkara bidentata* (773 years), *Ecclinusa guianensis* (448 years), *P. caimito* (240 years), and *Micropholis guyanensis* (248 years) (Laurance, Nascimento, et al. 2004). Consequently, the current adult tree cohort established before HP intensified in the Médio Juruá may persist for centuries, creating a significant lag before the demographic consequences of reduced seed dispersal can manifest at the community level. Additionally, we found that seed size was larger in scatter‐hoarded species and those dispersed through abiotic mechanisms. Our results therefore corroborate the notion that community‐wide variation in seed size may not be a strong indicator of the historical and contemporary effects of hunting on forest phytodemographics unless plant communities are teased apart by seed dispersal mode.

### 4.2 Interpreting the Timescale of Non‐Random Drift

Different timescales of analysis will affect the interpretation of how dispersal limitation may affect tree demography. For example, Hazelwood et al. (2020) examined the floristic transitions in tree plots in the Peruvian Amazon surveyed 14 years earlier by Terborgh et al. (2008). They reported that even when community composition diverged between persistently hunted and nonhunted sites, these changes could not be attributed to seed dispersal mode alone and argued that some saplings could have been recruited before the onset of commercial hunting in 1972 (Hazelwood et al. 2020). Estimating sapling age in tropical forests is at best difficult because juveniles can remain under arrested growth in the poorly illuminated understorey for many years (Green et al. 2014). Growth rates of Amazonian trees can range from 0.13 to 1.55 cm/year, which implies that most saplings would reach 5 cm DBH within 3–39 years, and only those at the slowest end of the spectrum would exceed 25 years (Reategui‐Betancourt et al. 2025). Growth rates are however highly light‐dependent, and without direct measurement of subplot canopy status, our ability to precisely attribute recruitment patterns solely to dispersal limitation, soil fertility, and water availability is constrained, as light availability is a key filter for sapling establishment (Reategui‐Betancourt et al. 2025). Yet, the 1–5 cm diameter saplings that we surveyed in this study are unlikely to be > 25 years old, which is approximately one‐quarter of the historical post rubber‐boom period in which hunters wielding fire‐weapons have operated most intensively in our Juruá study landscape.

Although this study is well‐replicated and represents incremental improvement in our understanding of the cascading effects of hunting on tropical forest floristic mosaics, longer‐term time series remain irreplaceable in elucidating cause–effect relationships (Hazelwood et al. 2020). However, reliably predicting tree community composition responses to hunting remains a huge challenge without a proper understanding of the associated mechanistic pathways. Outstanding uncertainties include the extent to which (1) the patterns uncovered here will either become stronger or weaker over time; (2) dispersal limitation of tree species associated with large‐bodied frugivores is the dominant driver of community‐wide juvenile recruitment; (3) surveyed saplings had indeed been recruited in the aftermath of the onset of regional‐scale intensive hunting; and (4) confounding effects of forest exploitation/degradation (e.g., timber extraction) can affect tree species associated with large‐bodied frugivores. Furthermore, while our use of genus‐level trait imputation for a minority of stems (10.8% for LMA and 21.5% for WD) is a conservative and phylogenetically justified approach (Aguirre‐Gutiérrez et al. 2025), we cannot entirely rule out the possibility that this introduced some degree of noise, potentially further contributing to the dilution of subtle community‐wide trait signals across the hunting gradient. Long‐term monitoring of permanent tree plots and their underlying conditions would shed light on all of these questions, including experimental studies with exclosures to control for seed predation and/or sapling herbivory.

### 4.3 Community‐Wide Functional Composition

Other plant functional traits that could be potentially indirectly affected by large‐bodied vertebrate declines include LMA and WD (Kurten et al. 2015). Based on the fast‐slow growth trade‐off along the plant economic spectrum (Reich 2014; Vaessen et al. 2023), we hypothesized that heavily hunted areas would favor lower LMA and WD due to the elevated recruitment of abiotically dispersed trees. Although we found a higher probability of sapling recruitment and elevated density of abiotically dispersed trees in the regeneration stratum of overhunted forests, this was not translated into divergent community‐wide LMA and WD values along the HP gradient as those reported by Vaessen et al. (2023). This likely results from the fact that we did not find significant differences in WD between abiotic and endozoochoric seed dispersal modes. Moreover, the most abundant species were tree species dispersed by non‐game or harvest‐insensitive game species, which have intermediate trait values (Hawes and Peres 2016). The lack of significant shifts in LMA or WD CWMs in saplings could be explained if the primary impacts of hunting, namely dispersal limitation and release from browsing pressure, are most pronounced at the seedling stage (Kurten et al. 2015; Wright, Stoner, et al. 2007; Vaessen et al. 2023). Thus, the initial demographic filters affecting seedling recruitment and survival may then be masked in the sapling cohort by later processes such as density‐dependent mortality, abiotic filtering, or a legacy effect from pre‐defaunation recruitment. In contrast, some heavy‐wooded, large‐seeded, and high leaf‐mass species—representing the slow woody biomass turnover—were almost exclusively dispersed by large‐bodied frugivores that are demonstrably sensitive to hunting (Hawes and Peres 2016; Scabin and Peres 2021). However, given that these species represented only ~2% of all 846 species inventoried or 2.2% of the overall BA across all 30 plots, their trait values became very diluted in community‐weighted means. Another possible explanation which has not been tested in our study landscape is the existence of compensation mechanisms, whereby the decline of one species is offset by the increase of another with similar functional traits. In the Médio Juruá region, defaunation may be causing a decline in highly vulnerable large‐seeded trees, but concurrently favoring the recruitment of other small‐seeded plant species dispersed by harvest‐insensitive generalist frugivores that have similar WD and LMA values.

The observed overall trend for higher average trait values in adult trees compared to saplings underneath can occur because, even if the understorey species composition reflects the adult tree layer (Wright et al. 2003), a limited light environment favors a higher proportion of light‐wooded saplings seeking a resource‐acquisitive strategy (Poorter and Bongers 2006). However, a higher prevalence of light‐wooded sapling species, compared with their co‐occurring conspecific trees, could only be detected in heavily hunted sites. Several environmental drivers such as enhanced CO_2_ fertilization can lead to a shift toward more acquisitive plant communities, and consequently, faster community dynamics (Brienen et al. 2015; Laurance, Nascimento, et al. 2004), but shifts observed here were associated only with sites experiencing heavy HP, which presumably overrode the potential effects of other concomitant drivers. Lower values of WD in juvenile tree composition can have a negative effect on the future carbon storage capacity of forests (Bello et al. 2015; Berenguer et al. 2018), since WD is an important predictor of aboveground biomass accumulation (Chave et al. 2006).

### 4.4 Implications for Conservation

This study sends a warning that even forests that have been moderately defaunated can show early signs of a hunting effect on forest composition and highlights the importance of understanding which plant–animal interactions are most affected by any given pattern of game harvest. This can inform sustainable game management initiatives in tropical forests that ensure retention of not only a full complement of forest vertebrate species, but also their key mutualistic interactions. We also highlight the need to monitor forest dynamics over time to understand whether these trophic cascades will be aggravated or mitigated in the long term.

Early signs of directional compositional change, however, were not necessarily reflected in the functional character of our forest sites. This is both because most mean trait values did not differ significantly between seed dispersal modes and the overwhelming influence of highly abundant species on community‐wide functional profiles, which were dominated by species dispersed by harvest‐insensitive frugivores.

## 5 Conclusions

Our findings suggest that persistently hunted forests, even in remote areas of the Amazon, show moderate signs of dispersal limitation as reflected in transitions in species taxonomic and functional composition as they transition from tree to sapling cohorts. However, these effects are apparently less pronounced than in studies elsewhere that only compare the opposite extremes of the HP gradient (e.g., nonhunted strictly protected areas vs. severely defaunated areas). Moderately defaunated tropical forests that still retain robust landscape connectivity with nonhunted areas, such as most of lowland Amazonia, may still retain most seed dispersal interactions even if key species of large‐bodied arboreal and terrestrial frugivores are kept below carrying capacity. This can be partly attributed to immigration of harvest‐sensitive species from adjacent nonhunted areas (Sirén et al. 2004; Levi et al. 2009) and a largely unknown degree of functional redundancy in dispersal interactions. In addition, some dispersal agents of large‐seeded plants often persist because they are either resilient to HP or ignored by hunters entirely (Peres and Palacios 2007). All of these factors may buffer the cumulative effects of game depletion on the floristic composition of hunted forests.

Nevertheless, sapling composition in increasingly hunted sites were on average less heavy‐wooded than their co‐occurring conspecific adult trees, which likely paves the way to future forest stands of lesser aboveground biomass and carbon stocks. These demographic transitions hold important implications for the ecosystem services provided by faunally depleted forests. This further justifies sustainable game management programs, which are still few and far between across tropical forests (Ingram et al. 2021; Sampaio et al. 2025) despite the fact that the spatial footprint of HP extends over ~29 mill. km^2^ (Philippe‐Lesaffre et al. 2025) and up to ~15 mill. km^2^ under risk of hunting‐induced defaunation (Benítez‐López et al. 2019; Ferreiro‐Arias et al. 2025).

## Author Contributions

**Andressa Bárbara Scabin:** conceptualization (lead), data curation (lead), formal analysis (equal), funding acquisition (lead), investigation (lead), methodology (lead), project administration (lead), resources (lead), validation (lead), visualization (lead), writing – original draft (lead), writing – review and editing (lead). **Iago Ferreiro‐Arias:** data curation (equal), formal analysis (lead), investigation (equal), methodology (equal), writing – original draft (equal), writing – review and editing (lead). **Flávia Regina Capellotto Costa:** data curation (equal), formal analysis (equal), investigation (equal), methodology (equal), writing – review and editing (equal). **Ana Benítez‐López:** data curation (equal), formal analysis (equal), investigation (equal), writing – original draft (equal), writing – review and editing (equal). **Cintia Gomes de Freitas:** formal analysis (equal), writing – review and editing (equal). **Carlos A. Peres:** conceptualization (equal), data curation (equal), formal analysis (equal), funding acquisition (equal), investigation (equal), methodology (equal), resources (equal), supervision (lead), writing – original draft (equal), writing – review and editing (equal).

## Funding

This work was supported by the National Geographic Society (grant number: 265943), Rufford Foundation (grant number: 21911‐1), Society for Conservation Biology [LACA Professional Development Award], DEFRA (Darwin Initiative for the Survival of Species) grant awarded to C.A.P. A.B.S. was awarded a PhD studentship from Coordenação de Aperfeiçoamento de Pessoal de Nível Superior—Brazil (CAPES) (Finance Code 001). This manuscript also acknowledges support from the TROPECOLNET project (ref. PID2022‐138272NA‐I00) funded by the Ministerio de Ciencia e Innovación, the Agencia Estatal de Investigación and FEDER (MCIN/AEI/10.13039/501100011033/FEDER, UE). C.A.P. is supported by a Frontiers Planet Prize awarded by the Frontiers Research Foundation.

## Conflicts of Interest

The authors declare no conflicts of interest.

## Supporting information


**Data S1:** ece372657‐sup‐0001‐supinfo.docx.

**Data S2:** ece372657‐sup‐0002‐supinfo.xlsx.

## Data Availability

All the required data are uploaded as Supporting Information.
