# Supplementary material for: Cascading Effects of Overhunting on the Functional Tree Composition of Amazonian Forests
Source: Ecol Evol. 2025 Dec 17;15(12):e72657. doi: 10.1002/ece3.72657 (PMC12710441; doi:10.1002/ece3.72657)
Supplement: Supplementary file 1 — Data S1: ece372657‐sup‐0001‐supinfo.docx. [file ECE3-15-e72657-s002.docx]

# Supplementary Information

## Supplementary Tables

**Table S1.** Number and percentage of stems with available trait values at species level or imputed at genus or family levels. SL: seed length, SW: seed width, SM: seed mass, LMA: leaf mass area, WD: wood density**.**

|  |  | **Abiotic** | | **Endozoochory** | | **Scatter-hoarding** | |
| --- | --- | --- | --- | --- | --- | --- | --- |
| **Trait** | **Imputation /Availability** | **N** | **(%)** | **N** | **(%)** | **N** | **(%)** |
| LMA | Family | 133 | 1.01 | 233 | 1.78 | 16 | 0.13 |
|  | Genus | 127 | 0.96 | 931 | 7.11 | 364 | 2.78 |
|  | Not available | 25 | 0.19 | 2291 | 17.5 | 614 | 4.69 |
|  | Species | 655 | 5 | 6554 | 50.04 | 1154 | 8.81 |
| Seed length | Family | 51 | 0.4 | 1229 | 9.51 | 0 | 0 |
|  | Genus | 682 | 5.28 | 5171 | 40 | 1136 | 8.79 |
|  | Species | 207 | 1.61 | 3438 | 26.6 | 1012 | 7.83 |
| Seed Mass | Family | 3 | 0.02 | 1380 | 10.77 | 0 | 0 |
|  | Genus | 498 | 3.89 | 5676 | 44.31 | 1619 | 12.64 |
|  | Not reported | 227 | 1.77 | 1106 | 8.64 | 50 | 0.39 |
|  | Species | 107 | 0.83 | 1664 | 12.99 | 479 | 3.74 |
| Seed width | Family | 51 | 0.4 | 1307 | 10.11 | 0 | 0 |
|  | Genus | 682 | 5.28 | 5003 | 38.7 | 1136 | 8.79 |
|  | Species | 207 | 1.61 | 3528 | 27.29 | 1012 | 7.83 |
| Wood Density | Family | 173 | 1.32 | 2513 | 19.19 | 76 | 0.58 |
|  | Genus | 80 | 0.61 | 1859 | 14.19 | 883 | 6.74 |
|  | Not available | 0 | 0 | 17 | 0.13 | 0 | 0 |
|  | Species | 687 | 5.24 | 5620 | 42.91 | 1189 | 9.08 |

**Table S2**. Taxonomic composition of trees ≥10 cm DBH and saplings (1-5 cm DBH), inventoried in 30 0.25-ha permanent tree plots and 30 0.05-ha sapling subplots in Médio Juruá region.

| **Family** | **Trees** | | **Saplings** | |
| --- | --- | --- | --- | --- |
|  | **Nº genera** | **Nº stems** | **Nº genera** | **Nº stems** |
| Achariaceae | 1 | 4 | 1 | 1 |
| Anacardiaceae | 3 | 28 | 4 | 19 |
| Anisophyllaceae | 1 | 1 | 1 | 4 |
| Annonaceae | 6 | 88 | 11 | 280 |
| Apocynaceae | 6 | 28 | 9 | 45 |
| Araliaceae | 1 | 9 | 2 | 19 |
| Arecaceae | 6 | 223 | 6 | 402 |
| Bignoniaceae | 2 | 13 | 2 | 5 |
| Bixaceae | 1 | 2 | 1 | 1 |
| Boraginaceae | 1 | 21 | 1 | 23 |
| Burseraceae | 5 | 221 | 5 | 418 |
| Caryocaraceae | 1 | 14 | 2 | 3 |
| Celastraceae | 1 | 2 | 1 | 3 |
| Chrysobalanaceae | 5 | 306 | 4 | 331 |
| Clusiaceae | 5 | 46 | 6 | 88 |
| Combretaceae | 1 | 9 | 2 | 3 |
| Connaraceae | 0 | 0 | 1 | 5 |
| Dichapetalaceae | 1 | 11 | 1 | 16 |
| Ebenaceae | 1 | 4 | 1 | 29 |
| Elaeocarpaceae | 1 | 61 | 1 | 107 |
| Erytrhoxylaceae | 1 | 1 | 1 | 15 |
| Euphorbiaceae | 17 | 341 | 14 | 340 |
| Fabaceae | 40 | 533 | 31 | 600 |
| Goupiaceae | 1 | 14 | 1 | 1 |
| Humiriaceae | 2 | 15 | 3 | 10 |
| Hypericaceae | 1 | 13 | 1 | 5 |
| Icacinaceae | 1 | 3 | 1 | 3 |
| Ixonanthaceae | 1 | 1 | 0 | 0 |
| Lacistemaceae | 1 | 2 | 1 | 5 |
| Lamiaceae | 1 | 8 | 1 | 3 |
| Lauraceae | 9 | 128 | 9 | 234 |
| Lecythidaceae | 6 | 714 | 6 | 201 |
| Lepidobotryaceae | 1 | 3 | 0 | 0 |
| Linaceae | 0 | 0 | 2 | 5 |
| Malpighiaceae | 2 | 3 | 1 | 4 |
| Malvaceae | 10 | 165 | 9 | 127 |
| Melastomataceae | 3 | 37 | 4 | 108 |
| Meliaceae | 3 | 83 | 2 | 258 |
| Menispermaceae | 0 | 0 | 1 | 1 |
| Monimiaceae | 1 | 3 | 1 | 16 |
| Moraceae | 10 | 276 | 9 | 383 |
| Myristicaceae | 3 | 384 | 4 | 367 |
| Myrtaceae | 5 | 48 | 5 | 126 |
| Nyctaginaceae | 2 | 46 | 2 | 95 |
| Ochnaceae | 2 | 8 | 2 | 34 |
| Olacaceae | 6 | 50 | 6 | 45 |
| Peraceae | 2 | 7 | 0 | 0 |
| Picramniaceae | 0 | 0 | 1 | 10 |
| Polygoniaceae | 1 | 3 | 1 | 1 |
| Proteaceae | 1 | 4 | 1 | 2 |
| Putranjivaceae | 1 | 13 | 1 | 2 |
| Quiinaceae | 1 | 2 | 1 | 12 |
| Rhizophoraceae | 2 | 8 | 2 | 4 |
| Rosaceaea | 1 | 1 | 0 | 0 |
| Rubiaceae | 12 | 46 | 15 | 115 |
| Rutaceae | 1 | 1 | 1 | 2 |
| Salicacea | 3 | 17 | 2 | 37 |
| Sapindaceae | 5 | 12 | 6 | 54 |
| Sapotaceae | 7 | 428 | 6 | 396 |
| Simaroubaceae | 1 | 13 | 1 | 15 |
| Siparunaceae | 1 | 24 | 1 | 187 |
| Solanaceae | 1 | 1 | 1 | 1 |
| Thymelaeaceae | 0 | 0 | 1 | 1 |
| Ulmaceae | 1 | 3 | 1 | 3 |
| Urticaceae | 2 | 114 | 2 | 90 |
| Violaceae | 3 | 50 | 4 | 385 |
| Vochysiaceae | 3 | 62 | 3 | 24 |
| Unidentified | 0 | 5 | 0 | 3 |
| Total | 227 | 4784 | 230 | 6132 |

|  |
| --- |
| **Table S3**. Mean and standard deviation of abundance and percentage of individual trees and saplings categorized by dispersal mode surveyed in 30 permanent tree plots of 0.25 hectares (DBH>10 cm) and 0.05 subplots (1-5 cm DBH) located along a hunting gradient in Médio Juruá region, Amazonas.   \|  \| **Abiotic** \| \| **Endozoochory** \| \| \| **Scatter-hoarding** \| \| \|  \| \| \| --- \| --- \| --- \| --- \| --- \| --- \| --- \| --- \| --- \| --- \| --- \| \| Adult \| Sapling \| \| Adult \| Sapling \| \| Adult \| Sapling \| \| \| **Abundance**  **(n ± SD)** \| 13.8 ± 5.3 \| 17.5 ± 9.7 \| \| 107 ± 21.4 \| 220.8 ± 48.4 \| \| 35.7 ± 14.4 \| 35.9 ± 14.3 \| \| \| **Percentage (% ± SD)** \| 8.6 ± 3.6 \| 6.4 ± 3.6 \| \| 67 ± 7.8 \| 79.3 ± 7.2 \| \| 22.4 ± 8.6 \| 13.3 ± 5.6 \| \| \|  \|  \|  \|  \|  \|  \|  \|  \|  \|  \| \|   **Table S4.** Summary of GLMM testing the effects of hunting pressure on the probability of sapling recruitment for plant species with different dispersal syndrome. CEC: cation exchange capacity, VDND: vertical distance to the nearest drainage. R^2^ conditional = 0.43 and R^2^ marginal = 0.15.   \| **Variable** \| **Estimate** \| **SE** \| **z value** \| **p-value** \| \| --- \| --- \| --- \| --- \| --- \| \| (Intercept) \| -0.386 \| 0.269 \| -1.433 \| 0.152 \| \| Dispersal mode (Endozoochory) \| 1.445 \| 0.276 \| 5.225 \| 0.000 \| \| Dispersal mode (Scatter-hoarding) \| 0.806 \| 0.356 \| 2.266 \| 0.023 \| \| Hunting pressure \| 0.055 \| 0.040 \| 1.371 \| 0.170 \| \| CEC \| -0.059 \| 0.041 \| -1.454 \| 0.146 \| \| VDND \| 0.012 \| 0.003 \| 3.749 \| 0.000 \| \| Number of conspecific adult trees \| -0.567 \| 0.036 \| -15.641 \| 0.000 \| \| Dispersal mode (Endozoochory):Hunting pressure \| -0.085 \| 0.042 \| -2.050 \| 0.040 \| \| Dispersal mode (Scatter-hoarding):Hunting pressure \| 0.004 \| 0.051 \| 0.080 \| 0.936 \|     **Table S5.** Summary of LMM testing the effects of hunting pressure on the sapling:tree abundance ratio for plant species with different dispersal syndrome. CEC: cation exchange capacity, VDND: vertical distance to the nearest drainage. R^2^ conditional = 0.21 and R^2^ marginal = 0.03.   \| **Variable** \| **Estimate** \| **SE** \| **t value** \| **p-value** \| \| --- \| --- \| --- \| --- \| --- \| \| (Intercept) \| -0.104 \| 0.219 \| -0.475 \| 0.635 \| \| Dispersal mode (Endozoochory) \| 0.210 \| 0.221 \| 0.950 \| 0.343 \| \| Dispersal mode (Scatter-hoarding) \| -0.185 \| 0.246 \| -0.750 \| 0.454 \| \| Hunting pressure \| 0.016 \| 0.034 \| 0.481 \| 0.630 \| \| CEC \| 0.029 \| 0.030 \| 0.970 \| 0.332 \| \| VDND \| 0.001 \| 0.002 \| 0.628 \| 0.530 \| \| Dispersal mode (Endozoochory):Hunting pressure \| -0.025 \| 0.035 \| -0.708 \| 0.479 \| \| Dispersal mode (Scatter-hoarding):Hunting pressure \| -0.015 \| 0.038 \| -0.393 \| 0.695 \|   **Table S6**. Summary of LMM testing the effect of hunting pressure on the sapling:tree abundance ratio for endozoochorous plant species with different seed length. CEC: cation exchange capacity, VDND: vertical distance to the nearest drainage. R^2^ conditional = 0.18 and R^2^ marginal = 0.014.   \| **Variable** \| **Estimate** \| **SE** \| **t value** \| **p-value** \| \| --- \| --- \| --- \| --- \| --- \| \| (Intercept) \| -0.023 \| 0.096 \| -0.239 \| 0.811 \| \| log10(Seed Length) \| 0.514 \| 0.307 \| 1.676 \| 0.094 \| \| Hunting pressure \| 0.015 \| 0.014 \| 1.083 \| 0.279 \| \| CEC \| 0.034 \| 0.034 \| 0.981 \| 0.327 \| \| VDND \| 0.002 \| 0.003 \| 0.832 \| 0.406 \| \| log10(Seed Length):Hunting pressure \| -0.113 \| 0.047 \| -2.422 \| 0.016 \|     **Table S7**. Summary of linear model testing the effects of hunting pressure on the CWM of wood density for trees and saplings. CEC: cation exchange capacity, VDND: vertical distance to the nearest drainage. R^2^ = 0.104.   \| **Variable** \| **Estimate** \| **SE** \| **t value** \| **p-value** \| \| --- \| --- \| --- \| --- \| --- \| \| (Intercept) \| 0.600 \| 0.010 \| 59.162 \| 0.000 \| \| Hunting pressure \| 0.003 \| 0.002 \| 1.719 \| 0.091 \| \| Life stage (juvenile) \| 0.010 \| 0.012 \| 0.789 \| 0.433 \| \| CEC \| 0.000 \| 0.004 \| -0.104 \| 0.918 \| \| VDND \| 0.001 \| 0.000 \| 1.624 \| 0.110 \| \| Hunting pressure:Life stage (juvenile) \| -0.003 \| 0.002 \| -1.263 \| 0.212 \|     **Table S8**. Summary of linear model testing the effects of hunting pressure on the CWM of leaf mass area for trees and saplings. CEC: cation exchange capacity, VDND: vertical distance to the nearest drainage. R^2^ = 0.11.   \| **Variable** \| **Estimate** \| **SE** \| **t value** \| **p-value** \| \| --- \| --- \| --- \| --- \| --- \| \| (Intercept) \| 0.019 \| 0.000 \| 57.916 \| 0.000 \| \| Hunting pressure \| 0.000 \| 0.000 \| 0.251 \| 0.803 \| \| Life stage (juvenile) \| -0.001 \| 0.000 \| -1.519 \| 0.135 \| \| CEC \| 0.000 \| 0.000 \| -0.200 \| 0.842 \| \| VDND \| 0.000 \| 0.000 \| 1.455 \| 0.151 \| \| Hunting pressure:Life stage (juvenile) \| 0.000 \| 0.000 \| 0.700 \| 0.487 \|   **Table S9**. Summary of linear model testing the effects of hunting pressure on the CWM of seed length for trees and saplings. CEC: cation exchange capacity, VDND: vertical distance to the nearest drainage. R^2^ = 0.54.   \| **Variable** \| **Estimate** \| **SE** \| **t value** \| **p-value** \| \| --- \| --- \| --- \| --- \| --- \| \| (Intercept) \| 1.267 \| 0.018 \| 69.176 \| 0.000 \| \| Hunting pressure \| 0.002 \| 0.003 \| 0.652 \| 0.517 \| \| Life stage (juvenile) \| -0.098 \| 0.022 \| -4.419 \| 0.000 \| \| CEC \| -0.006 \| 0.008 \| -0.864 \| 0.391 \| \| VDND \| 0.001 \| 0.001 \| 1.368 \| 0.177 \| \| Hunting pressure:Life stage (juvenile) \| 0.000 \| 0.004 \| 0.104 \| 0.918 \| |
|  |

## Supplementary Figures


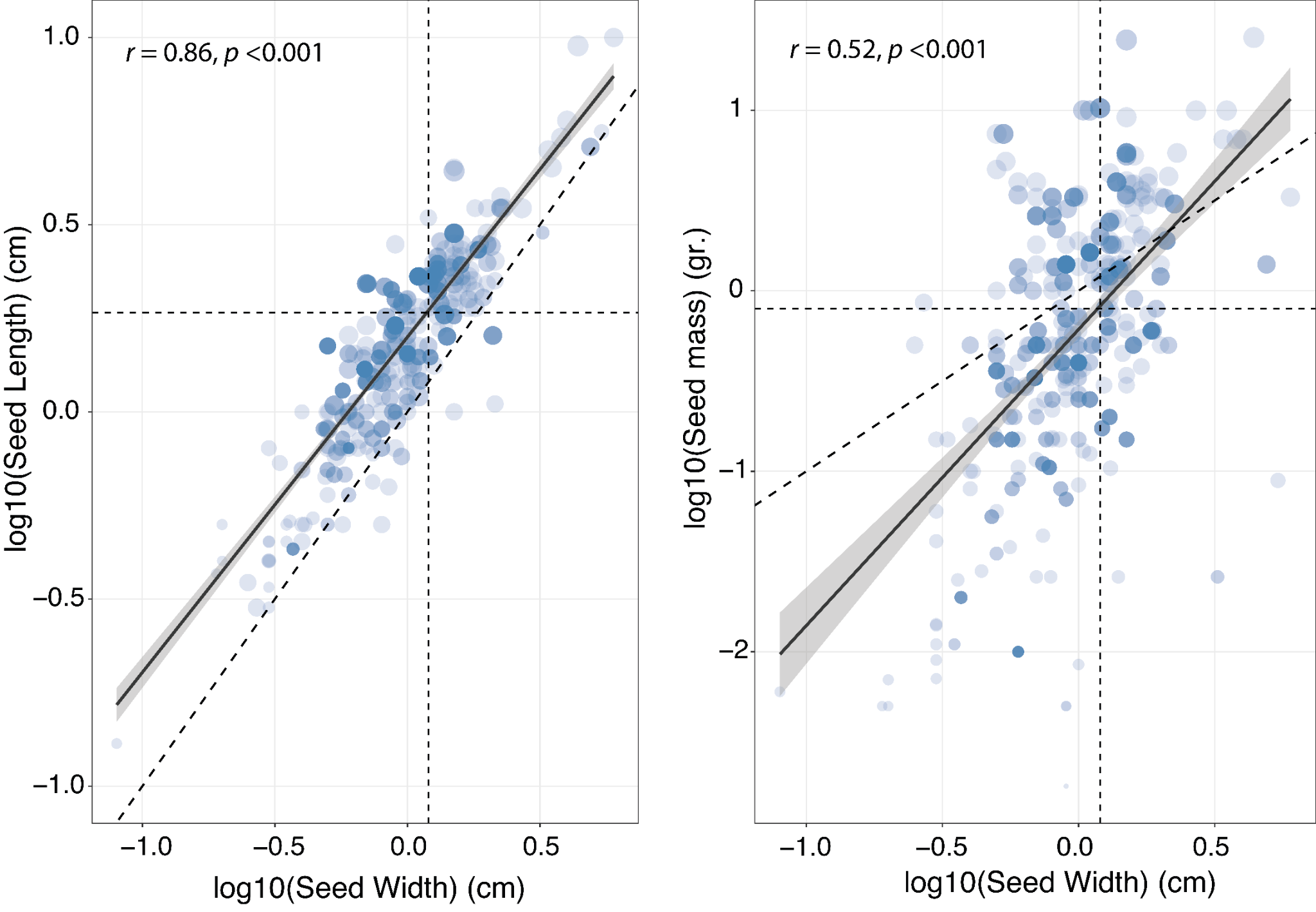


**Figure S1.** Correlation between seed width and seed length (left panel) and seed mass (right panel) of endozoochorous plant species, showing Pearson correlation coefficients (r) and significance values. Diagonal dashed line represents 1:1 relationship between seed length and seed width and seed length and seed mass respectively. Vertical lines represent the threshold of 12 mm of seed width, which has been previously related to a threshold for seed dispersal limitation (Galetti et al. 2013). Horizontal line represents the value of seed length or seed mass when seed width is equal to 12 mm.


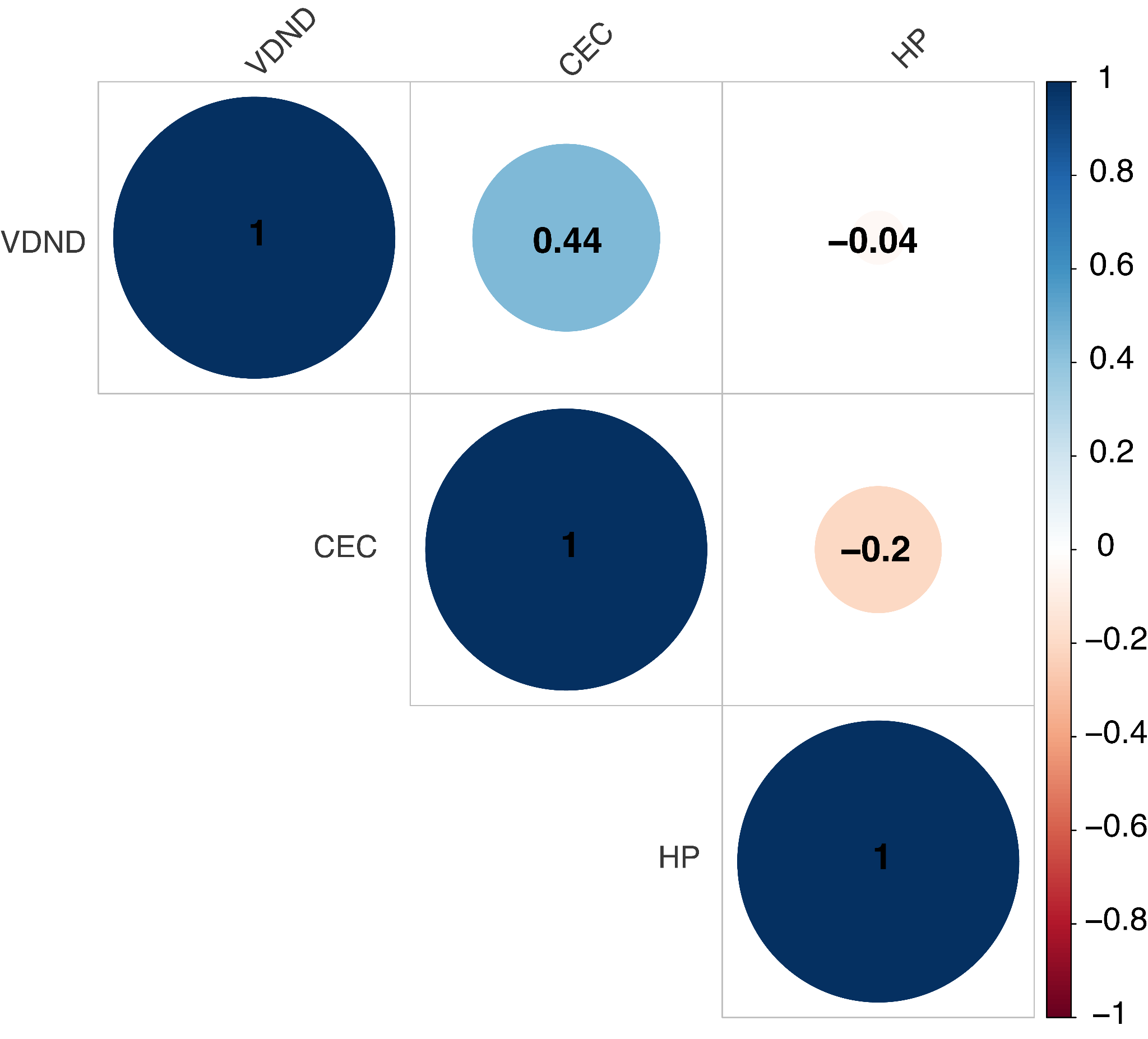


**Figure S2**. Multicollinearity assessment and Pearson correlation coefficients among predictors included to test hunting effects on probability of sapling recruitment, sapling:tree abundance ratio and community weighted means of plant traits. Predictor abbreviations: VDND (Vertical Distance to Nearest Drainage), CEC (Cation Exchange Capacity) and HP (Hunting Pressure).


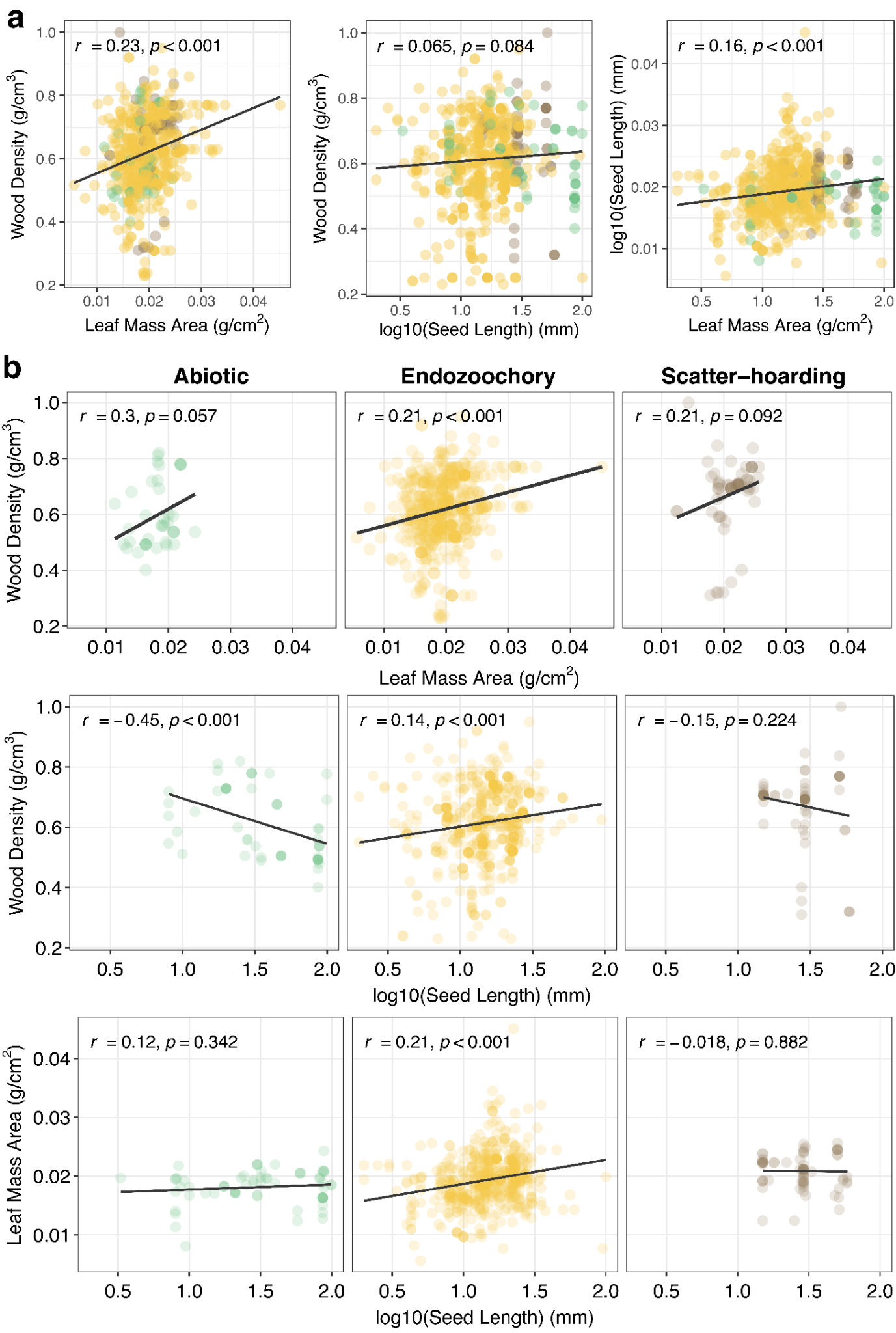


**Figure S3.** Relationship and Pearson correlation coefficients (r) between wood density, leaf mass per area and seed length for all plant species (Panel a) and for plants with different dispersal mode (Panel b): abiotically-dispersed, endozoochorous and scatter-hoarded plant species.
